# The Stanford Automated Mounter: pushing the limits of sample exchange at the SSRL macromolecular crystallography beamlines

**DOI:** 10.1107/S1600576716000649

**Published:** 2016-02-24

**Authors:** Silvia Russi, Jinhu Song, Scott E. McPhillips, Aina E. Cohen

**Affiliations:** a Stanford Synchrotron Radiation Lightsource / SLAC National Accelerator Laboratory, 2575 Sand Hill Road, MS 99, Menlo Park, California 94025, USA

**Keywords:** automated crystal mounting, high-throughput sample exchange, sample exchange robot, synchrotron robotics, protein crystallography, remote access, cryo-crystallography

## Abstract

The Stanford Automated Mounter System, a system for mounting and dismounting cryo-cooled crystals, has been upgraded to increase the throughput of samples on the macromolecular crystallography beamlines at the Stanford Synchrotron Radiation Lightsource. This upgrade speeds up robot maneuvers, reduces the heating/drying cycles, pre-fetches samples and adds an air-knife to remove frost from the gripper arms. Sample pin exchange during automated crystal quality screening now takes about 25 s, five times faster than before this upgrade.

## Abstract 2

The Structural Molecular Biology group at SSRL has upgraded its crystal mounting robot (SAM) to enable a sample pin exchange time of approximately 25 s during automated screening of samples.

## 1. Introduction   

The structural genomics and proteomics projects of the past decade have accelerated the development of automation at macromolecular crystallography beamlines, thereby enabling users of synchrotron beam time to collect and retrieve data at an ever increasing pace and efficiency (Abola *et al.*, 2000 ▸; Manjasetty *et al.*, 2008 ▸). During this time, diverse automated systems for mounting and dismounting crystals were designed and implemented at synchrotrons worldwide: ACTOR, CATS, ALS, SC3, MARCSC, G-Rob, SPACE and SAM (http://smb.slac.stanford.edu/robosync/). The Stanford Automated Mounter (SAM) was developed by the Structural Molecular Biology (SMB) group at the Stanford Synchrotron Radiation Lightsource (SSRL) (Cohen *et al.*, 2002 ▸). Automated sample handling with the SAM has had a tremendous impact, increasing the overall number of experiments carried out at SSRL and opening up the possibility for users (with cryo-cooled crystals) to conduct their experiments remotely while retaining full control of the SSRL macromolecular crystallography beamlines (McPhillips *et al.*, 2002 ▸; Soltis *et al.*, 2008 ▸; Smith & Cohen, 2008 ▸). Currently, more than 95% of SSRL macromolecular crystallography (MX) users at SSRL collect data remotely (*e.g.* from their home laboratories), saving time and avoiding travel costs. The reliability and reproducibility of the automated sample handling has not only increased crystal screening and data collection throughput, but also improved the quality of the diffraction data (Smith & Cohen, 2008 ▸). Moreover, the SAM system has been adopted at several synchrotrons sites: the Photon Factory in Tsukuba, Japan (Hiraki *et al.*, 2008 ▸), the National Synchrotron Radiation Research Center in Hsinchu City, Taiwan (Chao *et al.*, 2008 ▸), the Australian Synchrotron in Clayton, Australia (Cowieson *et al.*, 2015 ▸), the Canadian Light Source in Saskatoon, Canada (Grochulski *et al.*, 2012 ▸), and the Advanced Light Source in Berkeley, USA (Classen *et al.*, 2013 ▸). Recently, it has also been employed as the sample exchanger in frontier research applications for goniometer-based femtosecond crystallography at the XPP instrument of the Linac Coherent Light Source (Cohen *et al.*, 2014 ▸).

When the SAM system was put into service at the SSRL, the time frame needed for crystal centering and data collection was more commensurate with the time required for the SAM system to prepare itself for a mount/dismount cycle. Today, data sets may be collected in a few seconds, a result of an increase in X-ray source brightness and the adoption of a shutterless mode of data collection made possible by the introduction of fast frame rate X-ray area detectors such as the Dectris Pilatus 6M. Different approaches have been followed at different synchrotron sites to enhance the operation of the SAM system. A double-gripper design improved sample throughput (Hiraki *et al.*, 2008 ▸) and a larger liquid-nitrogen Dewar (Hiraki *et al.*, 2013 ▸) doubled the sample storage capacity of the system at the Photon Factory. Upgrades comprising an improved force sensing system have halved the time required for robot calibration and significantly increased the productivity at the Australian Synchrotron (T. Caradoc-Davies, oral presentation at the 2014 LCLS/SSRL Users’ Meeting). As described in the following sections, we also set out to upgrade SAM, a proven robust and versatile system, for a faster experimental pace.

## 2. Operation of the original SAM system   

**Figure 1:**
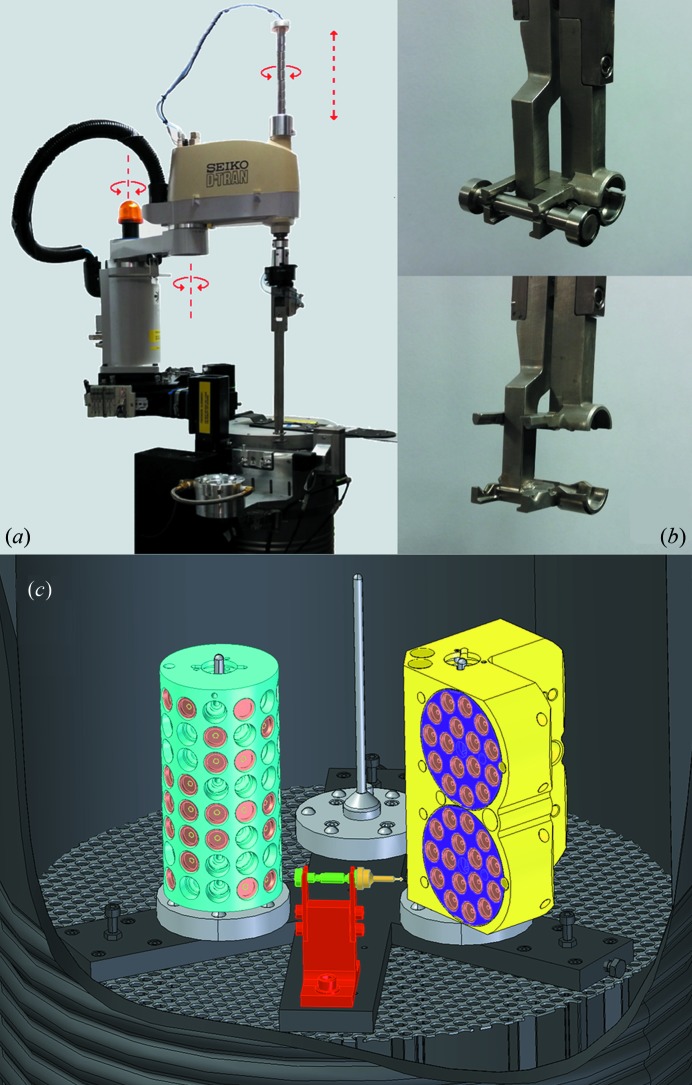
(*a*) Overall view of the SAM system. Arrows indicate the direction of motion of the robotic arm. (*b*) Detail of the gripper arm with the gripper in the closed position holding the dumbbell magnet tool (inset, top) and in the open position, empty (inset, bottom). (*c*) Cutaway of the Dewar showing a sample cassette (cyan), a puck adaptor (yellow) containing four uni-pucks (purple) and the loaded dumbbell magnet tool (green) resting on the cradle (red) with a sample pin (orange).

The original SAM system has been described in detail previously (Cohen *et al.*, 2002 ▸; Smith & Cohen, 2008 ▸). SAM is based on an off-the-shelf Epson E2S553S four-axis robot with a pneumatically activated metal gripper and an ATI force sensor outfitted on the translation shaft (Fig. 1 ▸ *a*). One side of the crystal transport gripper has small fingers used to hold a dumbbell-shaped magnetic tool and the opposite side has a cavity used to envelop the sample pin and maintain the crystal at cryogenic temperature as it is transferred to the goniometer. The dumbbell tool has two permanent magnets of different magnetic strength, with the stronger end (picker) designed to extract a sample pin from a storage cassette or uni-puck port, and the weaker end (placer) used to return a sample pin into a cassette or uni-puck port. The dumbbell magnet tool, either resting on a cradle or held by the gripper fingers (Figs. 1*b* and 1*c*), is always submerged within the liquid-nitrogen-filled storage Dewar that also holds up to three SSRL sample cassettes (for 288 sample pin capacity) or up to 12 uni-pucks (for 192 sample pin capacity).

During the original sample mounting process, the robot performed the steps of mounting in exactly the same way each time, with no optimizations or skipped steps. The mount cycle began with the robot gripper pulling a sample pin out of a cassette using the dumbbell magnet. Once the pin had been extracted, the gripper placed the dumbbell magnet tool into the cradle and released it, which allowed the gripper to rotate around and enclose the sample inside the gripper cavity. With the sample safely enclosed inside the gripper cavity, the robot pulled it off the dumbbell magnet and transferred the sample onto the goniometer magnet where it was released. While the sample was exposed to X-rays, the robot gripper went through a heat/drying and precooling cycle which required approximately two minutes to complete. Owing to the lock-step nature of the original process, the robot was required to wait for the drying cycle before reversing the process to dismount the sample. While loop centering and data collection could continue for part of this time, robot operations typically added 150 s for each sample. This full mount/dismount cycle had previously required a total of 3 min.

## 3. The upgraded SAM system   

To push the limits of robot operation to meet the demands of an ever increasing MX community, the SMB group has introduced new features to SAM and optimized the order and speed of robot motions without compromising the reliability and uptime of the system.

### 3.1. The robot motion speed   

For the original version of SAM, the velocity of robot motions was set at a regimen well below the apparatus’ maximum capabilities. For example, a speed of 200 mm s^−1^ was used for linear translations. Several tests were performed to determine the maximum speed of robot motions that would not compromise reliability. The robotic arm speed was incremented until the movements caused the liquid nitrogen to splash out of the Dewar. A regime just under this speed (of 800 mm s^−1^ for translations) was adopted during operations when the sample pin is inside the gripper cavity and inside the Dewar. When the sample pin is on the picker or placer magnet, an increase of velocity could cause damage to the sample; therefore, the original speeds were maintained for these operations. Outside the Dewar the maximum operational speed of the robot (1000 mm s^−1^ for translations) was implemented. The detector and beamstop are moved out of the way during sample exchange. However, the speed of the detector and beamstop positioners did not limit the rate of sample exchange and were not changed. The new faster-paced robot outperforms the old regime while maintaining reliability.

### 3.2. Reduced heating/drying cycles   

**Figure 2:**
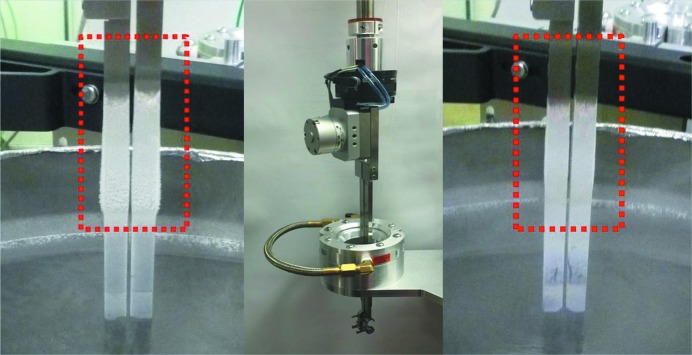
Frost formation and frost removal from the gripper arms. From left to right: ice formation on the grippers after 35 min of cooling within the liquid-nitrogen-filled Dewar, gripper arm inside the air-knife and the gripper arm after rapid translation through the air-knife.

The most time-consuming procedure in the crystal manipulation process was the lengthy heating/drying cycle that is necessary to eliminate condensation from the gripper and was performed each time a sample pin was handled. To optimize the performance of the robot, the number of heating/drying cycles was reduced. Tests were conducted to determine the maximum interval that the gripper could remain immersed in liquid nitrogen before ice formation on top of the gripper arms, just above the liquid-nitrogen bath, could compromise robot operation (Fig. 2 ▸). A worst-case situation was examined by performing tests at relative humidity levels up to 70%. Ideally, the longer the time interval between drying cycles the shorter overall sample-exchange time. However, a very long immersion resulted in the formation of ice between the two stainless steel arms of the gripper which could prevent the normal closing/opening of the gripper cavity and compromise robot operation. Therefore, the optimum interval was determined to be about 20 min. Although frost could accumulate on top of the gripper arms within 20 min, it did not solidify into a hard block or sheet. However, warming and eliminating this frost required a significantly longer heating cycle with the standard heater/drier system. To overcome this problem an air-knife (EXAIR Super-Air-Wipe #2402) was incorporated into the heating/drying process (see §3.3). The air-knife consists of a pair of semicircular halves with a number of compressed air outlets that join to form a uniform 360° ring of dry air focused into a cone just under the device. As the gripper is translated within the air-knife ring, pressurized air removes particles or moisture attached to its surface. The optimized gripper heating/drying protocol first uses the air-knife to blow frost off of the gripper arms; then the gripper enters the heater which melts any remaining frost; a second pass through the air-knife removes any excess liquid; and a final heating cycle removes any remaining humidity. Overall, the treatment takes 80 s (compared to the 40 s used previously). However, as the heating and cooling is now spread among multiple sample exchanges this represents a considerable time saving.

While skipping heating/drying cycles diminished the time burden by about 2 min per sample exchange, this new protocol requires measures to prevent any frost on the gripper arms from falling off and landing inside the gripper cavity where it could potentially damage the crystals. To prevent this, the gripper cavity remains closed when the robot is idle and the gripper is stored inside the Dewar filled with liquid nitrogen. The air-knife is then employed as a highly effective means to remove this frost from the upper part of the gripper arms. Before each mounting or dismounting operation, with the gripper cavity closed, the gripper is translated through the air-knife to remove any frost that has accumulated on the gripper arms, eliminating the chance that it could fall off into the gripper cavity when opened. This adds 5 s to each mount/dismount cycle.

### 3.3. Optimizing the order of robot operations   

**Figure 3:**
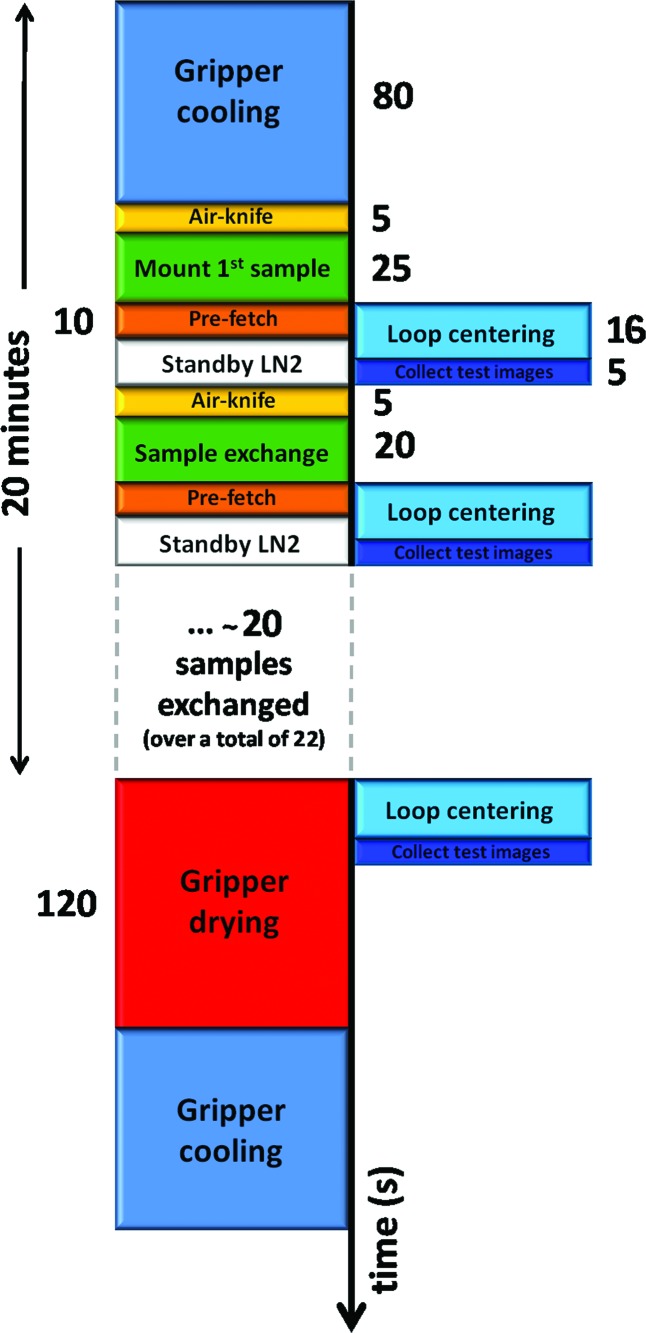
Timing diagram of the new SAM operation mode during sample screening.

From beginning to the end, the original robot sample exchange protocol, as described above in §2, only handles one sample pin at a time. The robot operations were reprogrammed, and in this new configuration, the robot prepares to mount the next sample pin while data collection is on-going (Fig. 3 ▸). During screening operations, the robot ‘pre-fetches’ the next sample in the queue using the picker magnet, which then is placed on the cradle waiting for the exchange. Once the current sample pin is ready to be dismounted, the gripper grabs the sample from the goniometer and puts it on the placer side of the dumbbell magnet tool. It then immediately goes to the picker, grabs the pre-fetched sample pin and mounts it onto the goniometer. By pre-fetching the next sample, 10 s are saved in the overall exchange process. Automated loop centering, data collection or other manual interventions by the user may continue as the gripper finishes the transfer of the first sample pin back into the cassette. The picker is then ready to pre-fetch the next sample and the process is repeated (Fig. 3 ▸).

**Figure 4:**
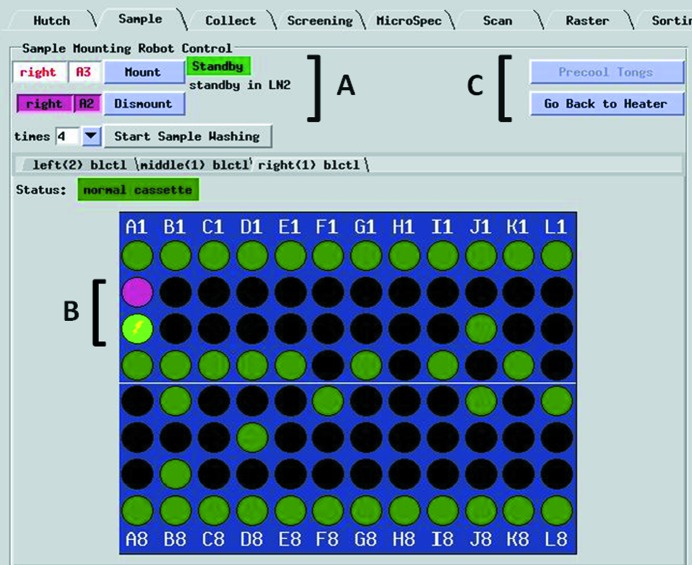
Detail of the *Blu-Ice* user interface highlighting the new features related to faster sample exchange. Control panel (A) showing the mounted sample, A2, highlighted in purple, and the selected sample to pre-fetch, A3. The gripper arm is in a ‘stand-by’ position within the liquid-nitrogen-filled dispensing Dewar. The currently pre-fetched and the mounted samples are displayed in the cassette diagram (B) in light green and purple, respectively. The lightning bolt indicates there is a sample queued (pre-fetched) for rapid sample exchange. Control panel buttons allow the user to pre-cool the gripper or to command it to the heater (C).

Sample pre-fetching has been incorporated into automated diffraction quality screening using *Blu-Ice*/*DCS* software. Pre-fetching may also be employed during semi-manual mounting using the *Blu-Ice* ‘Sample Tab’ (Fig. 4 ▸). After starting a sample mounting operation, the next sample to pre-fetch may be defined by clicking on the image of the cassette port. A lightning bolt will appear in the port image, as shown in Fig. 4 ▸. Using the pre-fetch mode the robot can reach a fast sample exchange that only takes 25 s.

## 4. Discussion and conclusions   

Since the beginning of the 2015 experimental run, the SAM systems at all SSRL macromolecular crystallography beamlines have been upgraded for faster operation. The system is about five times faster than the original version of SAM, taking on average 25 s to exchange a sample pin. To further improve the efficiency of crystal screening, efforts are underway to reduce the time required for automated centering of crystal-containing loops (Miller *et al.*, 2004 ▸; Soltis *et al.*, 2008 ▸). At SSRL BL12-2, the time required for automated loop centering has been reduced from over 35 s to 16 s by synchronizing the collection of video snapshots during sample rotation and by moving the sample translation stages simultaneously. Moreover, the loop-centering time will be further reduced through a planned upgrade of the SSRL micro-crystal goniometer to incorporate faster sample translation stages.

Although these developments will be important to enhance efficiency, even more effective measures to improve sample throughput, in particular for radiation-sensitive crystals that only survive a few X-ray exposures, will be the use of high-density sample containers that hold multiple crystals in known locations. These reduce the number of sample pins required for multi-crystal experiments and may be combined with technology for robotic sample mounting and automated sample positioning. Two examples of these technologies are micro-fluidic traps for micro-crystal data collection at room temperature (Lyubimov *et al.*, 2015 ▸) and the ‘sample mounting grid’ (Baxter *et al.*, 2016 ▸; Cohen *et al.*, 2014 ▸). The sample mounting grid holds of up to 75 conventionally sized crystals (100–300 µm), each within a small laser-cut hole, each hole equivalent to a single cryo-loop. The experimenter may align the grid one time, by clicking within a video display from within the *Blu-ice* control software, to initiate automated sequential positioning of multiple crystals into the X-ray beam position. Further developments underway at SSRL include support for fully automated room-temperature experiments through the use of humidity-controlled sample-storage enclosures in conjunction with a humidity-controlled air-stream at the goniometer.

In October 2014, the active community of SAM users and developers joined together in a workshop titled ‘Looking Ahead: SAM Developers Forum’, as part of the 2014 SSRL/LCLS Users’ Meeting. During the workshop it became clear that by working together improvements to the SAM system can be made more efficiently, so it was decided to make this a reoccurring event with the next workshop to be held at the Canadian Light Source.

## Supplementary Material

Robot Operation Video. DOI: 10.1107/S1600576716000649/te5009sup1.mpg

## References

[bb1] Abola, E., Kuhn, P., Earnest, T. & Stevens, R. C. (2000). *Nat. Struct. Biol.* **7**, 973–977. 10.1038/80754 11104004

[bb2] Baxter, E. L. *et al.* (2016). *Acta Cryst.* D**72**, 2–11.

[bb3] Chao, C.-H., Chiang, C.-H., Tseng, C.-C. & Jean, Y.-C. (2008). *Acta Cryst.* A**64**, C177.

[bb4] Classen, S., Hura, G. L., Holton, J. M., Rambo, R. P., Rodic, I., McGuire, P. J., Dyer, K., Hammel, M., Meigs, G., Frankel, K. A. & Tainer, J. A. (2013). *J. Appl. Cryst.* **46**, 1–13. 10.1107/S0021889812048698 PMC3547225 23396808

[bb5] Cohen, A. E., Ellis, P. J., Miller, M. D., Deacon, A. M. & Phizackerley, R. P. (2002). *J. Appl. Cryst.* **35**, 720–726. 10.1107/s0021889802016709 PMC4041710 24899734

[bb6] Cohen, A. E. *et al.* (2014). *Proc. Natl Acad. Sci. USA*, **111**, 17122–17127.

[bb7] Cowieson, N. P., Aragao, D., Clift, M., Ericsson, D. J., Gee, C., Harrop, S. J., Mudie, N., Panjikar, S., Price, J. R., Riboldi-Tunnicliffe, A., Williamson, R. & Caradoc-Davies, T. (2015). *J. Synchrotron Rad.* **22**, 187–190. 10.1107/S1600577514021717 PMC4294030 25537608

[bb8] Grochulski, P., Fodje, M., Labiuk, S., Gorin, J., Janzen, K. & Berg, R. (2012). *J. Struct. Funct. Genomics*, **13**, 49–55. 10.1007/s10969-012-9123-9 22270456

[bb9] Hiraki, M., Watanabe, S., pHonda, N., Yamada, Y., Matsugaki, N., Igarashi, N., Gaponov, Y. & Wakatsuki, S. (2008). *J. Synchrotron Rad.* **15**, 300–303. 10.1107/S0909049507064680 PMC2394784 18421164

[bb10] Hiraki, M., Yamada, Y., Chavas, L. M. G., Wakatsuki, S. & Matsugaki, N. (2013). *J. Synchrotron Rad.* **20**, 890–893. 10.1107/S0909049513021067 PMC3795550 24121334

[bb11] Lyubimov, A. Y., Murray, T. D., Koehl, A., Araci, I. E., Uervirojnangkoorn, M., Zeldin, O. B., Cohen, A. E., Soltis, S. M., Baxter, E. L., Brewster, A. S., Sauter, N. K., Brunger, A. T. & Berger, J. M. (2015). *Acta Cryst.* D**71**, 928–940. 10.1107/S1399004715002308 PMC4388268 25849403

[bb12] Manjasetty, B. A., Turnbull, A. P., Panjikar, S., Büssow, K. & Chance, M. R. (2008). *Proteomics*, **8**, 612–625. 10.1002/pmic.200700687 18210369

[bb13] McPhillips, T. M., McPhillips, S. E., Chiu, H.-J., Cohen, A. E., Deacon, A. M., Ellis, P. J., Garman, E., Gonzalez, A., Sauter, N. K., Phizackerley, R. P., Soltis, S. M. & Kuhn, P. (2002). *J. Synchrotron Rad.* **9**, 401–406. 10.1107/s0909049502015170 12409628

[bb14] Miller, M. D., Brinen, L. S., Cohen, A. E., Deacon, A., Ellis, P., McPhillips, S. E., McPhillips, T. M., Phizackerley, R. P., Soltis, S. M., van dem Bedem, H., Wolf, G., Xu, Q. & Zhang, Z. (2004). *Eighth International Conference on Synchrotron Radiation Instrumentation*, edited by T. Warwick, J. Stöhr, H. A. Padmore & J. Arthur, pp. 1233–1236. Melville: American Institute of Physics.

[bb15] Smith, C. A. & Cohen, A. E. (2008). *JALA*, **13**, 335–343. 10.1016/j.jala.2008.08.008 PMC2654326 19956359

[bb16] Soltis, S. M. *et al.* (2008). *Acta Cryst.* D**64**, 1210–1221. 10.1107/S0907444908030564 PMC2631117 19018097

